# A proposal for a new morphological classification of the popliteus muscle tendon with potential clinical and biomechanical significance

**DOI:** 10.1038/s41598-021-93778-5

**Published:** 2021-07-14

**Authors:** Łukasz Olewnik, Robert F. LaPrade, Friedrich Paulsen, Bartosz Gonera, Konrad Kurtys, Michał Podgórski, Paloma Aragonés, J. Ramón Sanudo, Michał Polguj

**Affiliations:** 1 Department of Anatomical Dissection and Donation, Medical University of Lodz, Lodz, Poland; 2 Twin Cities Orthopedics, Edina, MN USA; 3 Institute of Functional and Clinical Anatomy, Friedrich Alexander University Erlangen-Nürnberg, Erlangen, Germany; 4 Department of Topographic Anatomy and Operative Surgery, Sechenov University, Moscow, Russia; 5 Department of Imaging Diagnostic, Polish Mother’s Memorial Hospital Research Institute, Lodz, Poland; 6 Department of Orthopedics Surgery. Hospital Santa Cristina, Madrid, Spain; 7 Department of Human Anatomy and Embryology, Facultad de Medicina, Universidad Complutense de Madrid, Madrid, Spain; 8 Department of Normal and Clinical Anatomy, Chair of Anatomy and Histology, Medical University of Lodz, Lodz, Poland

**Keywords:** Anatomy, Musculoskeletal system, Muscle, Skeletal muscle

## Abstract

The purpose of this study was to characterize the morphological variations in the proximal attachments and create an accurate classification of the PPM for use in planning surgical procedures in this area, for evaluating radiological imaging and rehabilitation. One hundred and thirty-four lower limbs of body donors (52 woman and 82 man) fixed in 10% formalin solution were examined. The popliteus muscle was present in all 134 limbs. Four main types were identified with subtypes. The most common type was Type I (34.3%), characterized by a single tendon in the popliteus sulcus. Type II (30.6%) characterized by a main tendon in the popliteus sulcus and accessory bands. This type was divided into five subtypes (A–E) based on presence of specific accessory bands. Type III (15.3%) was characterized by two tendons in the popliteal sulcus. Type IV (19.4%) was characterized by two tendons in the popliteus sulcus and additional bands. This type was also divided into five subtypes (A–E) based on presence of specific accessory bands. The popliteofibular ligament was present in 90.3% of cases. A new classification based on a proximal attachment is proposed. The popliteus tendon is characterized by a very high morphological variability, which can affect posterolateral knee stability and the natural rotation of the tibia. Such a classification system may be useful for clinicians performing medical procedures within the knee joint, including orthopedic surgeons.

## Introduction

The posterolateral corner of the knee consists of the iliotibial band, long and short head of the biceps femoris muscle, fibular collateral ligament, posterior capsule, popliteus tendon (PLT), popliteofibular ligament (PFL), fabellofibular ligament and lateral coronary ligament^1–8^. The PLT runs posteriorly, medially and inferiorly across the femur, between the fibrous and synovial capsules of the knee joint, and leaves the posterior aspect of the joint. Following this, the tendon emerges from the knee joint and expands to form a flattened, triangular muscle. The belly of the muscle forms the lower part of the floor of the popliteal fossa, and it inserts into much of the posterior surface of the tibia above the soleal line^9^.

The popliteus muscle laterally rotates the femur on the tibia during the part of the gait cycle where one foot is on the ground, thus unlocking the knee; it also medially rotates the tibia on the femur when the involved limb is off the ground. Interestingly, of the muscles in the posterior compartment of the lower leg, it is the only one that acts on the knee and not the ankle. Together with the fibular collateral ligament and PFL, the PLT has a key role in the static stabilization of the posterolateral corner of the knee joint^1–4,10–16^.

The normal anatomy of the posterolateral aspect of the knee has been described in several studies^1–4,10–16^; however, the degree of morphological variability in this region remains unclear. The lateral and posterolateral knee region is well known to display high rate of morphological variations regarding the proximal and distal attachments of the ligaments^17,18^, relationships between the knee joint structures and the occurrence of additional bands^19,20^.

Although the posterolateral corner of the knee is infrequently injured in isolation, multiple ligament injuries can be caused by both sports injuries and high-energy trauma and severe disability can occur because of the knee instability and secondary degeneration of the articular cartilage^1^. Problems with the posterolateral corner also occur in conjunction with posterior cruciate ligament ruptures; co-occurrence of these injuries is observed in up to 60% of patients^1,2^. Reconstruction of the posterior cruciate ligament alone has been reported to be insufficient to restore normal activity^1,2^.

The purpose of this study was to characterize the morphological variations in the PPM proximal attachment and create an anatomic-based classification for use in planning surgical procedures in this area, radiological imaging and rehabilitation. The four main types will be useful to all clinicians (I-IV), while the subtypes are very important anatomically.

## Material and methods

### Anatomical study

One hundred and thirty-four lower limbs of body donors/cadavers (52 women and 82 men) fixed in 10% formalin solution were examined. The mean age “at death” of the cadavers was 75.1 years (38–93). The cadavers were the property of the Department, following donation to the university anatomy program. Any lower limbs with evidence of surgical intervention in the dissected area were excluded. All dissection of the leg and foot area was performed in accordance with the pre-established protocol^17–23^.

Dissection began with the removal of the skin and superficial fascia from the area of the knee and leg, up to the gastrocnemius muscle. Following this, the lateral and medial head of the proximal part of the gastrocnemius muscle were isolated from each other. The medial head of the gastrocnemius muscle was then partially removed, and the lateral head of the gastrocnemius muscle was separated at the musculotendinous junction, thus exposing the proximal part of the soleus muscle and plantaris muscle. The knee joint capsule was then thoroughly dissected, as well as the PPM and all structures around it^11,18,20,21,22,24^.

Upon dissection, the presence or absence of the PPM and the type of origin were recorded, and morphometric measurements of the PPM were taken.

#### When dissecting or operating the PPM, special care should be taken in the following cases

1. *Care must be taken when cleaning the posterior surface of the capsule of the knee joint to avoid removing the band connecting the capsule with the PPM.*
2. *After confirming that there is no connection between the PPM and the knee joint capsule, the capsule should be carefully severed, and the meniscus and posterior meniscus-femoral ligament checked to ensure that they are not connected.*
3. *Care should be taken not to cut a double tendon which may have been present in the proximal part, near the popliteal sulcus.*

All measurements were performed using an electronic digital caliper (Mitutoyo Corporation, Kawasaki-shi, Kanagawa, Japan). Each measurement was carried out twice by two researchers with an accuracy of up to 0.1 mm. There were two of the same researchers in two facilities (previously a research protocol was established). As confirmation of the first method, an additional measurement method was performed using the MultiScanBase 18.03 (Computer Scanning System II, Warsaw, Poland). The value and precision of this method have been confirmed in a previous study^17,18,20,25–29^.

### Histological study

After the PPM and popliteo-fibular ligament was collected, specimen were fixed in buffer (10% aqueous formalin) and then embedded in paraffin wax blocks. 8–10 µm thick histological slices were cut with a microtome (Semiautomatic microtom SLEE CUT5062, OPTA-TECH, made in Germany) and stained with hematoxylin and eosin and by Azan’s method to highlight the basic features of tissue architecture. Stained PPM and PFL slices were photographed using an Olympus CX43 (Olympus Corporation Japan) light microscope and an Olympus EP50 camera (Olympus Corporation Japan). Pictures were taken 2 × and 400 × and 600 × magnifications.

### Statistical analysis

Tendon types were compared between genders and laterality using the Chi^2^ test. The normality of the continuous data was tested using the Shapiro–Wilk test. As the distribution of the data was not normal, nonparametrical tests were used: morphological measurements between genders and bodysides were compared with the Mann–Whitney U-test, and measurements between popliteus tendon types with the Kruskal–Wallis test by ranks with a dedicated post hoc test.

All calculations were performed with Statistica 13.0 (Statsoft, Cracow). A *p* value lower than 0.05 was considered significant, with Bonferroni correction for multiple testing. The results are presented as mean and standard deviation, unless otherwise stated.

The Bioethics Committee of the Medical University of Lodz (resolution RNN/297/17/KE) approved the study protocol. The cadavers belong to the Department of Anatomical Dissection and Donation of the Medical University of Lodz, Poland, and to the Donors and Dissecting Rooms Center, Universidad Complutense de Madrid, Spain. Fifty limbs came from the Universidac Complutense de Madrit (14 women, 11 men), while the rest from the Medical University of Łódź.

### Classification—the principle of creation

The classification was based on specific types and subtypes.Types were labelled based on the number of bands found in the popliteal sulcus—(Type I and II had one main band in the popliteal sulcus, while Type III and IV had two main bands). For the purpose of creating a correct and clear classification, Type II and IV were divided into subtypes based on the number of additional bands and a site of their exact attachment.

The methods and all were carried out with relevant guidelines and regulations strictly developed and provided by Medical University of Lodz. The protocol of the study was accepted by Bioethics Committee of Medical University of Lodz (resolution RNN/297/17/KE). The cadavers belong to the Medical University of Lodz.

## Results

### Classification of the PLT

**Figure 1:**
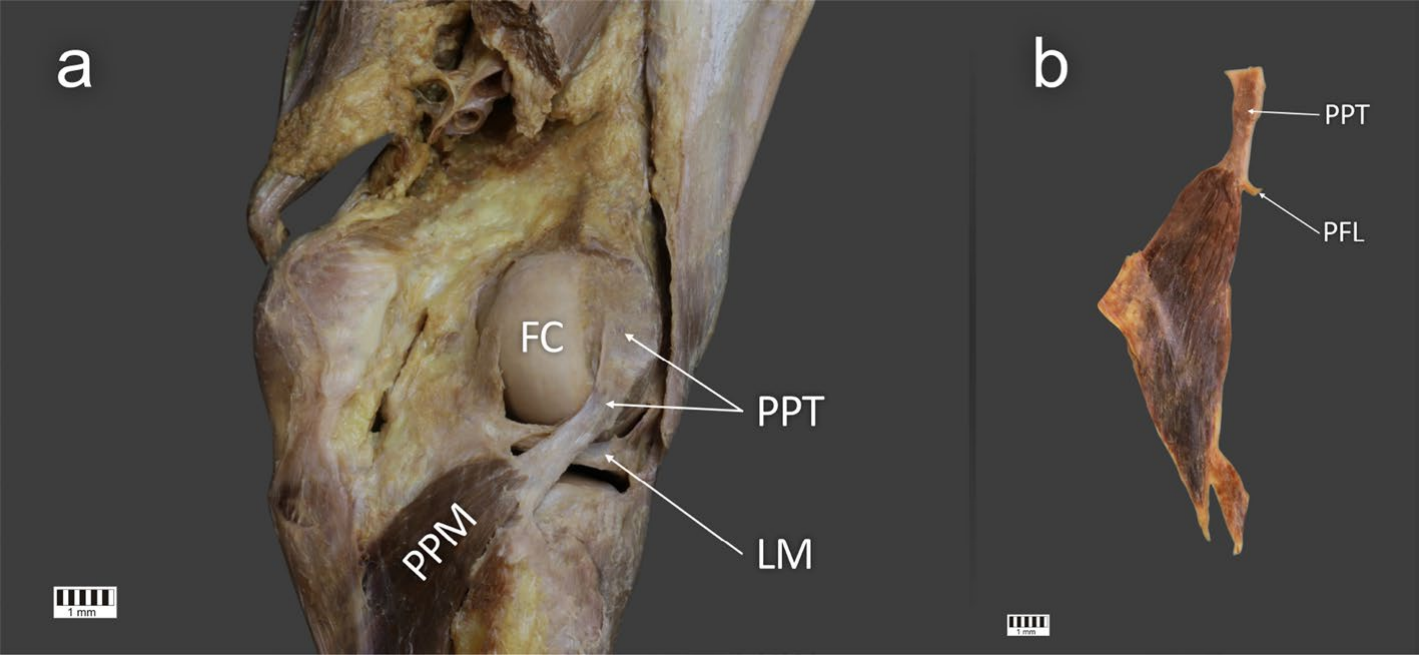
Type I of the popliteus tendon attachment. (**a**) Posterior view of the right knee joint with Type I of the popliteus tendon. (**b**) Isolated popliteus muscle with Type I of the popliteus tendon. PFL—popliteofibular ligament, PPT—popliteus tendon, FC—femoral condyle, LM—lateral meniscus PPM—popliteus muscle. The Fig. 1 was taken with Canon EOS 6D camera with Canon EF 100 mm f/2.8 L Macro Is USM, and the signatures were made in Corel DRAW 12 (https://www.coreldraw.com/pl/pages/coreldraw-12/).

**Figure 2:**
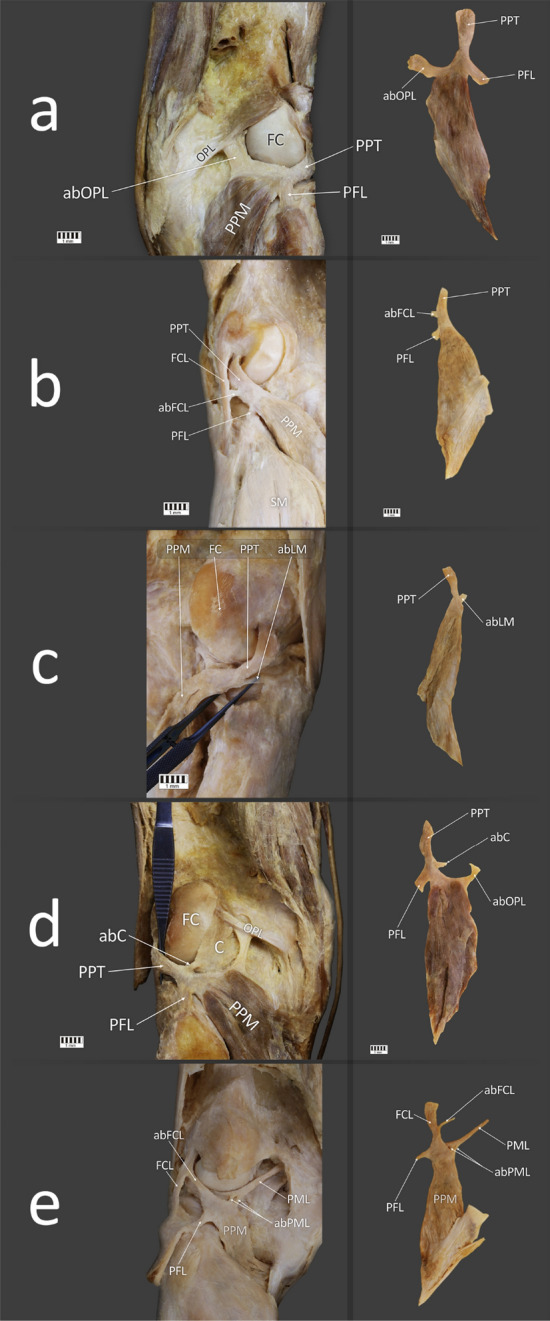
Type II of the popliteus tendon attachment. (**a**) LEFT—Posterior view of the right knee joint with Type IIa of the popliteus tendon. RIGHT—Isolated popliteus muscle with Type IIa of the popliteus tendon. (**b**) LEFT—Posterior view of the left knee joint with Type IIb of the popliteus tendon. RIGHT—Isolated popliteus muscle with Type IIb of the popliteus tendon. (**c**) LEFT—Posterior view of the right knee joint with Type IIc of the popliteus tendon. RIGHT—Isolated popliteus muscle with Type IIc of the popliteus tendon. (**d**) LEFT—Posterior view of the left knee joint with Type IId of the popliteus tendon. RIGHT—Isolated popliteus muscle with Type IId of the popliteus tendon. (**e**) LEFT—Posterior view of the left knee joint with Type IIe of the popliteus tendon. RIGHT—Isolated popliteus muscle with Type IIe of the popliteus tendon. PFL—popliteo-fibular ligament, PPT—popliteus tendon, PPM—popliteus muscle, OPL—oblique popliteal ligament, abOPL—accessory band to the oblique popliteal ligament, FC—femoral condyle, FCL—fibular collateral ligament, abFCL—accessory band to the fibular collateral ligament, SM—soleus muscle, abLM—accessory band to the lateral meniscus, C—the knee joint capsule, abC—accessory band to the knee joint capsule, PML—posterior meniscofemoral ligament, abPML—accessory band to the posterior meniscofemoral ligament. The Fig. 2 was taken with Canon EOS 6D camera with Canon EF 100 mm f/2.8 L Macro Is USM, and the signatures were made in Corel DRAW 12 (https://www.coreldraw.com/pl/pages/coreldraw-12/).

**Figure 3:**
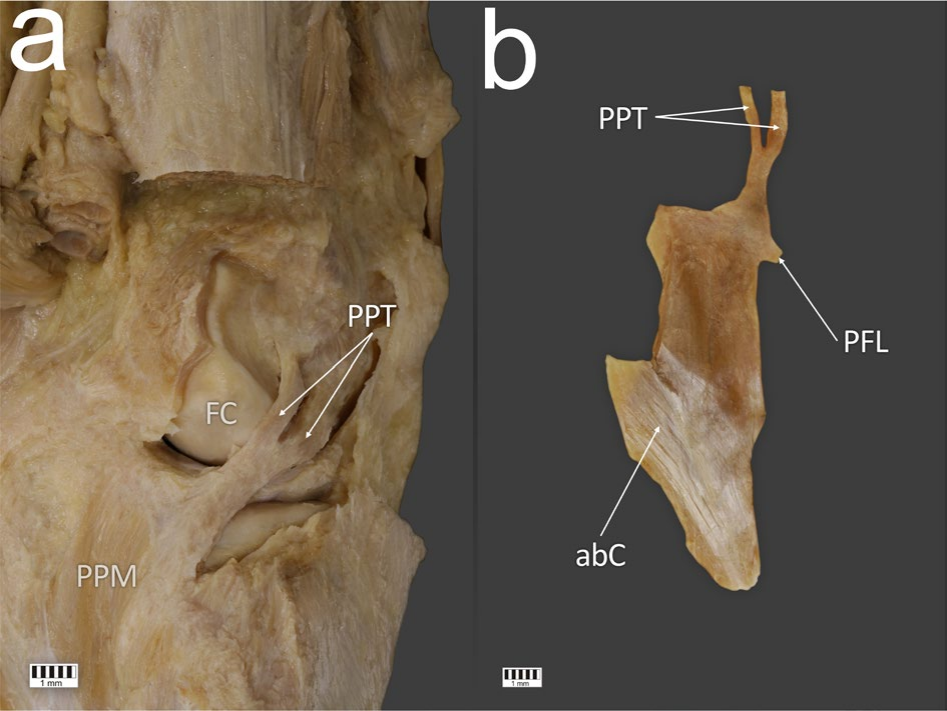
Type III of the popliteus tendon attachment. **a** Posterior view of the right knee joint with Type III of the popliteus tendon. **b** Isolated popliteus muscle with Type III of the popliteus tendon. FC—femoral condyle, PPT—popliteus tendon, PPM—popliteus muscle, PFL—popliteofibular ligament, abC—accessory band to the knee joint capsule. The Fig. 3 was taken with Canon EOS 6D camera with Canon EF 100 mm f/2.8 L Macro Is USM, and the signatures were made in Corel DRAW 12 (https://www.coreldraw.com/pl/pages/coreldraw-12/).

**Figure 4:**
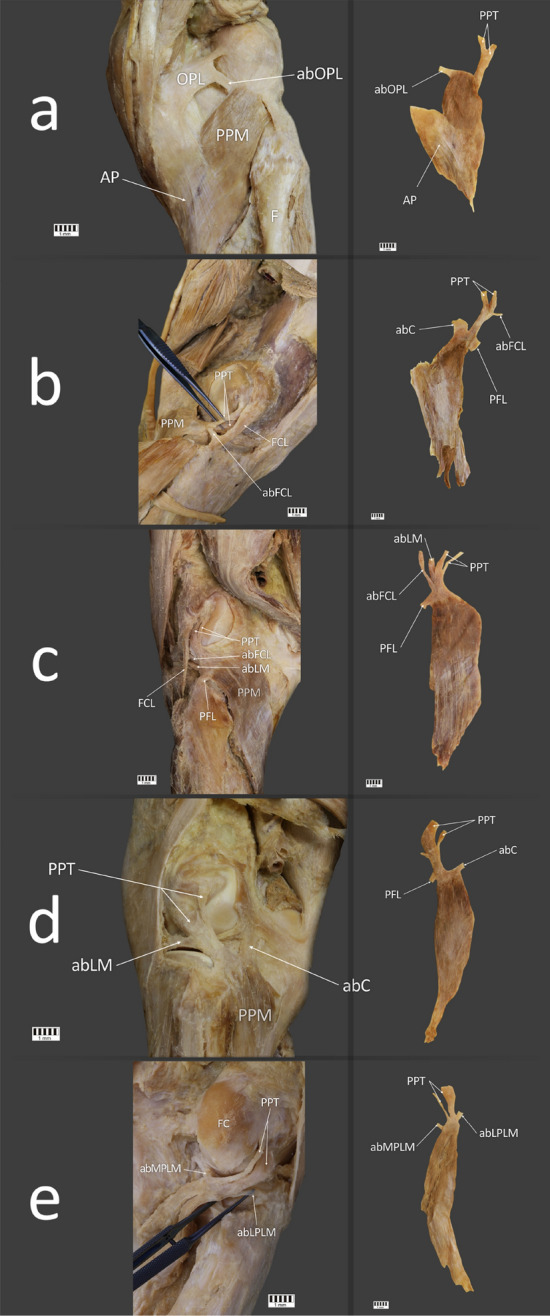
Type IV of the popliteus tendon attachment. (**a**) LEFT—Posterior view of the right knee joint with Type IVa of the popliteus tendon. RIGHT—Isolated popliteus muscle with Type IVa of the popliteus tendon. (**b**) LEFT—Posterior view of the right knee joint with Type IVb of the popliteus tendon. RIGHT—Isolated popliteus muscle with Type IVb of the popliteus tendon. (**c**) LEFT—Posterior view of the left knee joint with Type IVc of the popliteus tendon. RIGHT—Isolated popliteus muscle with Type IVc of the popliteus tendon. (**d**) LEFT—Posterior view of the left knee joint with Type IVd of the popliteus tendon. RIGHT—Isolated popliteus muscle with Type IVd of the popliteus tendon. (**e**) LEFT—Posterior view of the right knee joint with Type IVe of the popliteus tendon. RIGHT—Isolated popliteus muscle with Type IVe of the popliteus tendon. F—fibula, PFL—popliteo-fibular ligament, PPT—popliteus tendon, PPM—popliteus muscle, OPL—oblique popliteal ligament, abOPL—accessory band to the oblique popliteal ligament, FC—femoral condyle, FCL—fibular collateral ligament, abFCL—accessory band to the fibular collateral ligament, abLM—accessory band to the lateral meniscus, abC—accessory band to the knee joint capsule, AP—aponeurosis, abMPLM—accessory band to the medial part of the lateral meniscus, abLPLM—accessory band to the lateral part of the lateral meniscus. The Fig. 4 was taken with Canon EOS 6D camera with Canon EF 100 mm f/2.8 L Macro Is USM, and the signatures were made in Corel DRAW 12 (https://www.coreldraw.com/pl/pages/coreldraw-12/).

The PPM was present in all 134 limbs. The following types were differentiated based on morphological features:

- Type I—a single tendon attached to the popliteal sulcus (proximal half of the sulcus). This type was found in 46 lower limbs (34.3%) (Fig. 1a, b).
- Type II—(41 limbs; 30.6%) a single tendon attached to the popliteus sulcus. The main tendon inserted as in Type I; however, the following subtypes were introduced according to the attachment of the accessory bands:
  - Type IIa—a single accessory band attached to the oblique popliteal ligament (17 limbs) (Fig. 2a).
  - Type IIb—a single accessory band attached to fibular collateral ligament (12 limbs) (Fig. 2b).
  - Type IIc—a single accessory band attached to the lateral part of the lateral meniscus (6 limbs) (Fig. 2c).
  - Type IId—two accessory bands were present: the first attached to the posterior articular capsule and the second to the oblique popliteal ligament (4 limbs) (Fig. 2d).
  - Type IIe—three accessory bands were present: the first attached to the fibular collateral ligament and the second and third attached to the posterior meniscofemoral ligament (2 limbs) (Fig. 2e).
  - Type III—two tendons were present in the popliteal sulcus (21 limbs; 15.7%) (Fig. 3a, Fig. 3b).
- Type IV—(26 limbs; 19.4%) two tendons were present in the popliteal sulcus, the main tendons inserted as in Type III; however, the following subtypes were introduced according to the attachment of the accessory bands:
  - Type IVa—the accessory band attached to the oblique popliteal ligament (5 limbs) (Fig. 4a).
  - Type IVb—two accessory bands were present: the first attached to the fibular collateral ligament and the second attached to the posterior articular capsule (4 limbs) (Fig. 4b).
  - Type IVc—two additional bands were present: attaching to the fibular collateral ligament and the lateral meniscus (7 limbs) (Fig. 4c).
  - Type IVd—two additional bands were present: attaching to the lateral part of the lateral meniscus and the posterior articular capsule (6 limbs) (Fig. 4d).
  - Type IVe—two additional bands were present: attaching to the lateral part of the lateral meniscus and the medial part of the lateral meniscus (4 limbs) (Fig. 4e).

**Figure 5:**
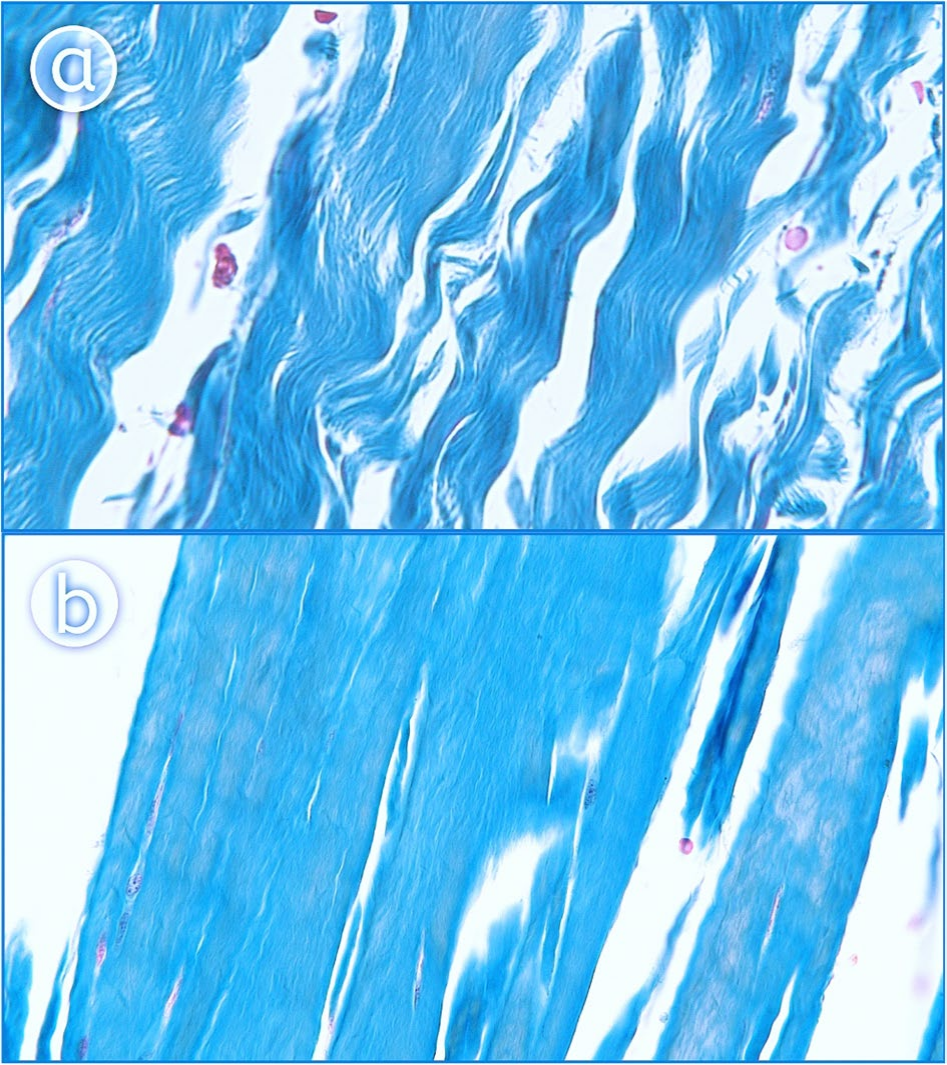
Histological examination of the accessory bands of the popliteus muscle. A 400 magnification—ligament. B 400 × magnification—tendon. The Fig. 5 was taken with Olympus EP50 camera (Olympus Corporation Japan), and the signatures were made in Corel DRAW 12 (https://www.coreldraw.com/pl/pages/coreldraw-12/).

The PFL was not considered in the classification due to being a ligamentous structure (Fig. 5a). The other bands (to the posterior capsule, to the fibular collateral ligament, to the lateral meniscus, to the meniscofemoral ligament) had tendinous texture in histological examination (Fig. 5b).

The numbers of each type according to sex and the body side are presented in Table 1.

**Table 1:** The number of specified types depends on the sex and the side of the body.

Type	Total number	Male	Female	Right side	Left side
I	46	29	17	21	25
IIa	17	9	8	9	8
IIb	12	9	3	6	6
IIc	6	3	3	3	3
IId	4	4	0	2	2
IIe	2	0	2	1	1
III	21	12	9	11	10
IVa	5	3	2	3	2
IVb	4	4	0	2	2
IVc	7	5	2	4	3
IVd	6	4	2	3	3
IVe	4	4	0	2	2

No significant difference in the frequency of tendon types was observed between the body sides (*p* = 0.9999) and sexes (*p* = 0.1108). Morphometric measurements are presented in Supplementary Table 1 according to body sides/sexes, and in Supplementary Table 2 according to tendon type.

### Aponeurosis of the PPM

The aponeurosis of the PPM merged with fibres of tendon of the semimembranosus muscle and oblique popliteal ligament (Fig. 4a) in 134 specimens.

**Figure 6:**
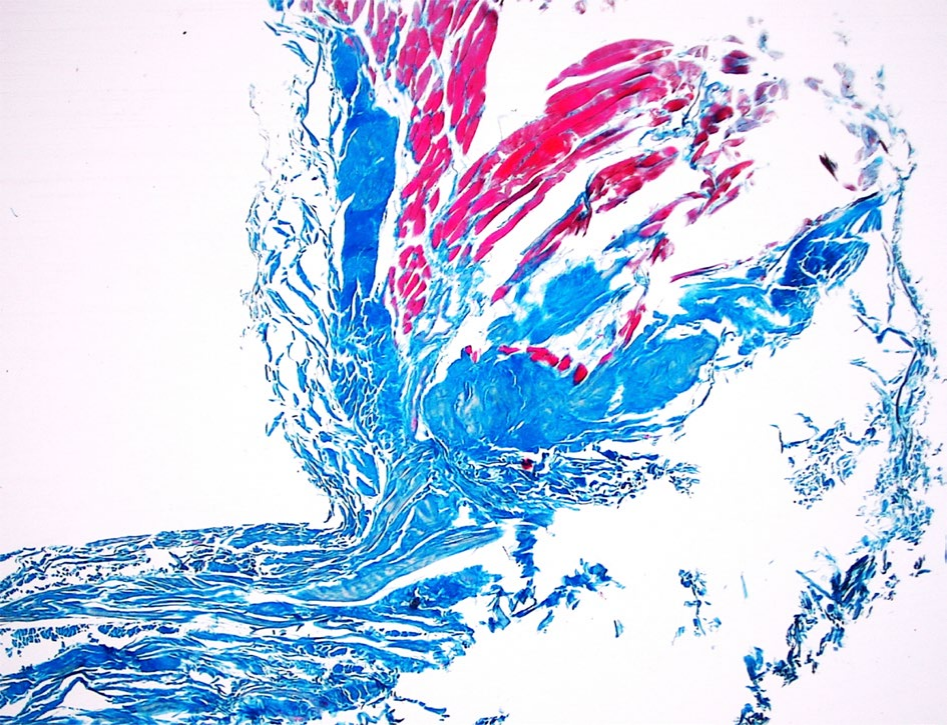
Histological examination of the aponeurosis of the popliteus muscle—2 × magnification. The Fig. 6 was taken with Olympus EP50 camera (Olympus Corporation Japan).

In histological terms, aponeurosis looks like a sheetlike fibrous membrane, histologically similar to tendon, where the parallel bundles of collagen exist as multiple layers alternating at 90° angles to one another. Our histological findings confirm that the fibre system correspond to an aponeurosis (Fig. 6.) The aponeurosis of the PPM differed significantly between different types of tendon, with regard to width at the beginning of the aponeurosis (*p* = 0.0001); width at the distal attachment (*p* = 0.0001); and thickness at distal attachment (*p* = 0.0106) (Supplementary Table 1).

### Popliteofibular ligament (PFL)

The PFL was found in 121 lower limbs (90.3%).

### Ethical approval and consent to participate

The study protocol was accepted by the Bioethics Committee of the Medical University of Lodz (resolution RNN/297/17/KE). The cadavers were the property of the Department of Anatomical Dissection and Donation, Medical University of Lodz, and of the Donors and Dissecting Rooms Center, Universidad Complutense de Madrid, Spain.

### Consent to publish

Not applicable.

## Discussion

The most important finding of the present work was that it comprehensively analysed the quantitative anatomy of the PLT and presents a systematic classification of the PPM origin based on anatomical dissection and that it includes new categories.

Relatively little is known about the accessory bands of the PPM. In addition, no classification of the PPM exists which would assist in planning of surgical procedures within the knee area, especially in the posterolateral aspect. Previous anatomical studies on cadavers have only described their variability in relation to the distance between the fibular collateral ligament and popliteus tendon attachment, as well as the relationship between them^1,4,11,30^. Alternatively, several studies so far have attempted to specify the possible types of proximal attachment of tendon of the PPM, but they were carried out on very small samples and are still not systematized^11,12^. Feipel et al.^24^ only described sites of the tendon attachment of the PPM; posterior capsule, arcuate popliteal ligament, oblique popliteal ligament, fibula, lateral meniscus, posterior cruciate ligament and posterior meniscofemoral ligament. In contrast, Jung et al.^11^ classified three types of the PPM tendon attachment based on a relationship with the FCL: posteroinferior, just inferior and a double attachment by bifurcated bands.

Chuncharunee et al.^12^ distinguished four types: the first type (13.7%) had only one attachment at the lateral femoral condyle, while the second and third type had two attachments. The lateral femoral condyle and posterior horn of the lateral meniscus constituted the attachments for the second type (34.2%), while the lateral femoral condyle and fibular head for the third type (23.3%). The fourth type (28.8%) was characterized by three attachments: the lateral femoral condyle, the posterior horn of the lateral meniscus and the fibular head^12^. The origin of the PPM is an important taxonomic category; however, it is possible that the number of specimens used in previous studies was too limited to reveal all of the most important morphological variations due to the considerable tendency to vary inherent in the PPM morphology.

We propose a four-fold classification of the PPM origin based on specific types and subtypes. Types were created based on the number of bands found in popliteal sulcus—(Type I and II have one main band in popliteal sulcus, while Type III and IV have two main bands). To improve accuracy and clarity, Type II and IV have been divided into subtypes based on the number of additional bands and the site of their exact attachment. The PFL was not considered in the classification due to the fact it had ligamentous texture; histological examination found the other bands to have tendinous texture.

The most common type was Type I (34.3%), which was characterized by the attachments to the popliteus sulcus (proximal half of the sulcus). This type of popliteus tendon attachment was characterized by the widest attachment of all types, with a mean width of 8.67 mm (*p* = 0.0001). This type may have the strongest effect on the stabilization of the posterolateral aspect of the knee joint and is extremely important for potential knee ligament reconstruction (PCL and FCL). In contrast, Type III (15.7%), characterized by two tendons in the popliteal sulcus, was the least common type. This type of split in the popliteal sulcus can reduce posterior tibial translations and lower the forces acting on the posterior cruciate ligament. This type of attachment may be particularly important for posterior cruciate ligament reconstructions. Of the remaining types, Type II (30.6%) and Type IV (19.4%), were characterized by additional single and double accessory bands with no attachments in popliteal sulcus. These accessory bands can have very important clinical and biomechanical significance.

Single accessory bands were found in Type IIa and IVa. The presence of an accessory band attached to the popliteal oblique ligament suggests that the PPM helps prevent hyperextension in the knee and excessive external rotation. In addition, the oblique popliteal ligament has an attachment located on semimembranosus muscle, suggesting that the PLT may affect the semimembranosus muscle.

On the other hand, the presence of an accessory band to the fibular collateral ligament (Type IIb) suggests that popliteus muscle helps the fibular collateral ligament to resist a range of forces at all knee flexion angles; However, when at an angle in the range 0°–30°, it appears to resist tension, and counter external rotation when near maximum extension^17,18,31–34^. As a result of different sorts of trauma and hyperextension, the lesion of the fibular collateral ligament occurs. After a hard impact to the medial side of the leg, it is most commonly injured, but a lesion may occur in this same movement pattern after a heavy hit to the lateral portion of the foot or a non-contact injury^18,35^. Therefore, it ought to be considered whether such a connection between the fibular collateral ligament and the PLT will prevent such injuries. A significant feature of fibular collateral ligament injuries is that they rarely occur as single incidents; they typically co-occur with knee joint area injury to other ligaments, such as the anterior cruciate ligament, posterior cruciate ligament, and other posterolateral corner structures^36^. To do damage simultaneously to all the above-mentioned structures (multi-ligament knee injury), a large amount of force is needed, such as a high-impact sports collision; a question arises whether the PLT often ruptures as the fibular collateral ligament does.

One of the most interesting connections was noticed in Type IIc where an accessory band of the PLT attached to the lateral meniscus. What does this connection mean? Will the PLT support the lateral meniscus? In some way, will the PPM help in disperse weight of a body and reduce friction due to its contraction during movement? Meniscal tears are the most frequently encountered and treated injuries concerning the knee joint, with a bimodal age distribution in young, active sports people and in elderly people. Similarly, meniscal tear surgery is counted in the most commonly performed procedures in orthopedic surgery^8^. Can this connection somehow protect the lateral meniscus injury?

The remaining types are characterized by the presence of more than one additional band (Type IId, IIe IVb, IVc, IVd, IVe).

In the present research, the posterior aspect of the knee joint capsule was also found as one of possible attachment sites of the PLT. As a rule, they were very wide attachments, which may suggest that PLT supports and protects posterior capsule of the knee joint with inferior side. Interestingly, the second range of attachments was the popliteal oblique ligament (Type IId). It should be considered that this Type can affect both lateral meniscus and popliteal oblique ligament. From the biomechanical point of view, this variant would be very supportive for both the meniscus and the ligament, however from the immunological point of view it may cause an inflammation as plantaris does in mid-portion Achilles tendinopathy^20,23,25–27,37–40^. Types: IVb, IVc, IVd, IVe are a combination of the above-described potential influences. The last type to describe is Type IIe, which is characterized by three additional bands (first to the fibular collateral ligament, second and third to the posterior meniscofemoral ligament). This type of an attachment may indicate how important the popliteus muscle is in the posterolateral corner of the knee. Both the interactions of the fibular collateral ligament and the posterior cruciate ligament via the posterior meniscofemoral ligament may be critical for orthopedic surgeons operating on the posterior cruciate ligament and fibular collateral ligament ruptures^3,5,13,18,36,41–43^.

The present research shows that it is pointless as previously thought to describe the association between the fibular collateral ligament attachments and the PLT, due to too great morphological variability and in many cases the PLT is combined with fibular collateral ligament via additional bands^1,4,11,31^. Previous studies included a very small research sample, hence probably why the authors did not observe such significant morphological differences^1,4,11,31^.

It is reported that posterolateral knee corner injuries constitute almost 16% of all knee injuries and, if not diagnosed properly, are responsible for sustained instability and failure of concomitant reconstructive surgery^5,6^. Reports also indicated that to ensure anatomical reconstruction of an avulsed structure, the attachment site (or sites) of the avulsed ligament or tendon must be identified. It can be difficult and challenging, particularly in cases of chronic posterolateral corner injury, to locate the attachment sites of the fibular collateral ligament, PLT, PFL, and biceps femoris tendon during surgery. The challenge of locating and recognizing the typical/original attachment sites of these structures can be enhanced by retraction and scarring.

In the present study, we have shown that the PFL is not a permanent structure; it is present in 90.3% of all limbs. Due to that a question arises; what is the impact of its lack on the functioning of the posterolateral corner of the knee? In the present study, we localized the aponeurosis of the PPM, which merges with the fibres of the tendon of the semimembranosus muscle and oblique popliteal ligament. The incidence of aponeurosis was constant—100%.

There are some limitations to this research. A greater sample size would have been ideal, but the small size (n = 134) was attributed to the morphological diversity of the muscle. Nevertheless, the study has a significantly greater sample size and reveals substantial differences relative to prior research^11,12,24^. In addition, the sample population was recruited from a particular population of people in the area around Lodz, Poland and around Madrid, Spain who have spent the better part of their lives. Therefore, to reveal whether the observed morphological variations are present in larger populations, more comprehensive studies are required. Furthermore, no sample size measurement was conducted; However, it should be noted that this research is one of the largest so far conducted on the morphological variants of popliteus tendon attachments. Furthermore, the accurate attachment(s) and accessory bands have not been researched in previous studies; however, a different attachment classification should be used. Nevertheless, our work is the first of this scale to reveal and describe an entirely new classification of the attachment of the popliteus tendon. Such a formal classification would definitely be a powerful instrument for improving the results of future popliteal region treatments.

## Conclusion

The popliteus tendon is characterized by the great morphological variability. Accordingly, the introduction of a new classification based on its proximal attachment and presence of additional bands is necessary. The PFL prevalence was 90.3% while the aponeurosis was present in 100%. Different types of the popliteus tendon attachment and its additional bands may impose on researchers some new clinical and biomechanical issues. Such a systematic classification can be valuable for clinicians who perform medical procedures within the knee joint area.

## Supplementary Information

Supplementary Information 1.

Supplementary Information 2.

## Data Availability

Please contact authors for data requests (Łukasz Olewnik PhD–email address: lukasz.olewnik@umed.lodz.pl).
