# Supplementary material for: A proposal for a new morphological classification of the popliteus muscle tendon with potential clinical and biomechanical significance
Source: Sci Rep. 2021 Jul 14;11:14434. doi: 10.1038/s41598-021-93778-5 (PMC8280136; doi:10.1038/s41598-021-93778-5)
Supplement: Supplementary file 1 — Supplementary Information 1. [file 41598_2021_93778_MOESM1_ESM.docx]

Table 1. Morphometric parameters according to sex and bodyside (mm).

| Parameter | Sex | | P value | Body side | | P value |
| --- | --- | --- | --- | --- | --- | --- |
|  | Females | Males |  | Right | Left |  |
| Muscle belly length | 97.07 (7.95) | 101.18 (10.05) | 0.0137 | 99.07 (9.83) | 100.11 (9.16) | 0.5280 |
| Main tendon length | 32.49 (5.58) | 37.09 (6.20) | 0.0001 | 35.80 (6.30) | 34.82 (6.42) | 0.4918 |
| Main tendon width (proximal) | 7.02 (2.61) | 7.42 (2.72) | 0.4099 | 7.38 (2.76) | 7.15 (2.61) | 0.6692 |
| Main tendon thickness (proximal) | 2.00 (0.97) | 2.19 (0.99) | 0.3564 | 2.09 (1.01) | 2.14 (0.97) | 0.6245 |
| Musculotendinous junction width | 8.04 (2.59) | 8.21 (2.06) | 0.4665 | 8.08 (2.20) | 8.21 (2.35) | 0.7758 |
| Musculotendinous junction thickness | 2.67 (1.09) | 2.37 (0.88) | 0.1446 | 2.53 (1.01) | 2.44 (0.93) | 0.4988 |
| Width at the beginning of the aponeurosis | 13.76 (3.78) | 14.92 (3.97) | 0.1080 | 14.31 (3.84) | 14.64 (4.03) | 0.7403 |
| Thickness at the beginning of the aponeurosis | 1.51 (0.76) | 1.87 (0.75) | 0.0071 | 1.71 (0.76) | 1.75 (0.79) | 0.7554 |
| Width at the aponeurosis (distal attachment) | 15.05 (3.69) | 16.27 (4.97) | 0.2289 | 15.58 (4.62) | 16.01 (4.50) | 0.5616 |
| Thickness at the aponeurosis (distal attachment) | 1.63 (0.77) | 1.79 (1.06) | 0.4906 | 1.74 (1.01) | 1.72 (0.90) | 0.7656 |
| Second tendon length | 18.33 (7.37) | 24.95 (14.61) | 0.2551 | 21.95 (12.70) | 22.65 (12.65) | 0.7735 |
| Second tendon width (proximal) | 4.44 (2.23) | 3.93 (1.02) | 0.5804 | 4.28 (1.95) | 3.98 (1.16) | 0.8146 |
| Second tendon thickness (proximal) | 1.20 (0.40) | 1.62 (0.95) | 0.1525 | 1.41 (0.84) | 1.49 (0.77) | 0.4302 |
| Second tendon width (distal) | 4.48 (1.91) | 3.40 (1.20) | 0.0579 | 3.97 (1.80) | 3.69 (1.36) | 0.7899 |
| Second tendon thickness (distal) | 1.18 (0.55) | 1.12 (0.65) | 0.3513 | 1.13 (0.58) | 1.17 (0.64) | 0.8646 |
| First additional band length | 11.27 (5.33) | 9.85 (5.51) | 0.1960 | 10.53 (5.79) | 10.26 (5.12) | 0.8556 |
| First additional band width (proximal) | 2.47 (0.81) | 3.52 (1.87) | 0.0269 | 3.09 (1.56) | 3.14 (1.71) | 0.8018 |
| First additional band thickness (proximal) | 1.17 (0.51) | 1.32 (0.96) | 0.9692 | 1.19 (0.87) | 1.33 (0.76) | 0.1939 |
| First additional band width (distal) | 2.31 (0.91) | 3.48 (2.17) | 0.0302 | 3.15 (2.18) | 2.89 (1.48) | 0.9500 |
| First additional band thickness (distal) | 0.92 (0.53) | 0.98 (0.85) | 0.5889 | 0.92 (0.76) | 1.00 (0.73) | 0.5552 |
| Second additional band length | 14.63 (8.43) | 15.08 (7.43) | 0.8408 | 14.69 (7.54) | 15.14 (8.08) | 0.8083 |
| Second additional band width (proximal) | 2.54 (0.94) | 3.33 (1.32) | 0.1027 | 2.92 (1.03) | 3.17 (1.46) | 0.8271 |
| Second additional band thickness (proximal) | 1.31 (0.81) | 1.48 (0.63) | 0.6879 | 1.39 (0.67) | 1.45 (0.74) | 0.7341 |
| Second additional band width (distal) | 2.20 (0.79) | 3.21 (1.75) | 0.0564 | 2.58 (0.89) | 3.11 (2.02) | 0.5604 |
| Second additional band thickness (distal) | 1.07 (0.88) | 1.20 (0.82) | 0.8018 | 1.09 (0.81) | 1.21 (0.87) | 0.6800 |
| Third additional band length | 3.35 (0.04) |  |  | 3.38 (-) | 3.32 (-) |  |
| Third additional band width (proximal) | 1.29 (0.01) |  |  | 1.28 (-) | 1.29 (-) |  |
| Third additional band thickness (proximal) | 0.42 (0.01) |  |  | 0.41 (-) | 0.43 (-) |  |
| Third additional band width (distal) | 1.50 (0.02) |  |  | 1.48 (-) | 1.51 (-) |  |
| Third additional band thickness (distal) | 0.47 (0.04) |  |  | 0.44 (-) | 0.49 (-) |  |

p-value lower than 0.0024 is significant according to Bonferroni correction.
