# Supplementary material for: A proposal for a new morphological classification of the popliteus muscle tendon with potential clinical and biomechanical significance
Source: Sci Rep. 2021 Jul 14;11:14434. doi: 10.1038/s41598-021-93778-5 (PMC8280136; doi:10.1038/s41598-021-93778-5)
Supplement: Supplementary file 2 — Supplementary Information 2. [file 41598_2021_93778_MOESM2_ESM.doc]

Table. 2. Morphometric parameters according to tendon types

| Parameter | Tendon types | | | | P value |
| --- | --- | --- | --- | --- | --- |
|  | I | II | III | IV |  |
| Muscle belly length | 99.76 (13.11) | 98.43 (7.64) | 99.93 (6.99) | 100.84 (5.60) | 0.7730 |
| Main tendon length | 35.28 (6.18) | 35.73 (6.54) | 31.68 (4.50) | 37.63 (6.68) | 0.0085 |
| Main tendon width (proximal) | 8.67 (3.18)* | 7.43 (1.84) | 5.54 (1.85) | 5.90 (1.91)* | 0.0001 |
| Main tendon thickness (proximal) | 2.21 (1.05) | 2.18 (0.90) | 1.87 (0.47) | 2.05 (1.28) | 0.5635 |
| Musculotendinous junction width | 8.70 (2.18) | 8.51 (2.84) | 7.16 (1.44) | 7.39 (1.45) | 0.0099 |
| Musculotendinous junction thickness | 2.68 (0.70) | 2.30 (0.63) | 2.74 (1.41) | 2.23 (1.29) | 0.0245 |
| Width at the aponeurosis beginning | 12.55 (4.20)* | 14.66 (3.61)* | 16.43 (3.89)* | 16.00 (2.16)* | 0.0001 |
| Thickness at the aponeurosis beginning | 1.74 (0.89) | 1.73 (0.83) | 1.58 (0.59) | 1.84 (0.56) | 0.4288 |
| Width at the aponeurosis distal attachment | 15.93 (4.80)* | 17.59 (4.83)* | 16.11 (3.14)* | 12.35 (2.34)* | 0.0001 |
| Thickness at the aponeurosis distal attachment | 2.10 (0.91) | 1.46 (0.88) | 1.65 (0.90) | 1.57 (1.04) | 0.0106 |
| Second tendon length |  |  | 14.13 (7.30) | 28.86 (12.09) | 0.0000 |
| Second tendon width (proximal) |  |  | 3.93 (0.73) | 4.30 (2.09) | 0.9318 |
| Second tendon thickness (proximal) |  |  | 1.24 (0.41) | 1.62 (0.99) | 0.1776 |
| Second tendon width (distal) |  |  | 3.56 (1.15) | 4.07 (1.88) | 0.4100 |
| Second tendon thickness (distal) |  |  | 0.90 (0.52) | 1.34 (0.61) | 0.0054 |
| Firs additional band length |  | 9.68 (4.02) |  | 11.54 (7,.08) | 0.8369 |
| Firs additional band width (proximal) |  | 3.19 (1.77) |  | 2.99 (1.38) | 0.9078 |
| Firs additional band thickness (proximal) |  | 1.20 (0.95) |  | 1.36 (0.56) | 0.1355 |
| Firs additional band width (distal) |  | 3.14 (1.92) |  | 2.84 (1.80) | 0.6112 |
| Firs additional band thickness (distal) |  | 0.97 (0.86) |  | 0.94 (0.50) | 0.5933 |
| Second additional band length |  | 12.82 (6.97) |  | 15.51 (7.90) | 0.3974 |
| Second additional band width (proximal) |  | 3.06 (1.87) |  | 3.03 (1.05) | 0.9767 |
| Second additional band thickness (proximal) |  | 0.93 (0.50) |  | 1.56 (0.69) | 0.0356 |
| Second additional band width (distal) |  | 2.93 (1.71) |  | 2.81 (1.53) | 0.8153 |
| Second additional band thickness (distal) |  | 0.59 (0.26) |  | 1.31 (0.86) | 0.0752 |
| Third additional band length |  | 3.35 (0.04) |  |  | - |
| Third additional band width (proximal) |  | 1.29 (0.01) |  |  | - |
| Third additional band thickness (proximal) |  | 0.42 (0.01) |  |  | - |
| Third additional band width (distal) |  | 1.50 (0.02) |  |  | - |
| Third additional band thickness (distal) |  | 0.47 (0.04) |  |  | - |

p-value lower than 0.0024 is significant according to Bonferroni correction. * - significant differences according to *post hoc* test.
